# Wounds of the Abdomen

**Published:** 1856-09

**Authors:** 


### *Wounds of the Abdomen.* By Dr. Robards.

I was called, during the coldest weather in December, in consultation with Dr. Peyton, of this place, to visit two men that had been severely wounded in a fight that took place on a Ferry Boat, while crossing Loosa Hatchie, about four miles above the city. On our arrival, we found both the patients in a small log house, occupied by the ferryman. One, Mr. B. was lying on the bed, bleeding profusely from various wounds in different parts of the body; the most serious of which, however, was one just above the elbow joint in front, which severed the whole of the integuments down to the bone. The other patient, Mr. S. was lying on the floor before a large fire, apparently in a dying condition. He also had received numerous wounds, inflicted with the same knife, which, though not seen by us, was supposed to be an ordinary large dirk knife, with a blade ¾ of an inch wide, and six or eight long. The wound in this case that was of the most importance, and seemed inevitably fatal, was in the abdomen—on the left side, a little below the umbilicus. A large amount of intestine and omentum was protruding, and resting upon the floor. It must have been inflicted at least an hour and a half before we saw it, and on inquiry we found that after receiving the wound he jumped into the river, and either swam or waded ashore, with this amount of intestine and omentum hanging out (it could not have been less than a hat-full,) and the thermometer standing considerably below freezing point. On reaching the shore, he laid some time on a log, and was then taken into the house by some friends. Being in a state of collapse when I examined him, pulse feeble, the extremities cold and clammy, the features contorted and palid; he was let alone to die, and our attention directed to Mr. B., whose wound occupied ¾ of an hour in dressing. In the meantime, however, S. frequently called for aid, begging us to give him morphine and let him die easy, that he knew he was obliged to die, &c. He was given large doses, but no brandy—he had taken quite enough of that before the fight. Finally after becoming a little excited and relieved by the morphine, he began to beg to be dressed, and said no one knew but that he might get well yet. I asked him many questions while still engaged in dressing the wounds of the other patient, among them was, what kind of a pistol he shot his adversary with. He replied, "a d—d good one." (I mention this fact to show what powerful influence the mind and nerve can exercise in a case of severe wounds of any kind.) In my opinion, nine-tenths of the men in the world would have died of similar wounds and under similar circumstances, in three hours.

Finally, my attention was directed to the dressing of his wounds, more for decency's sake than with any hope of saving him. The intestines, though wounded in various places, had only one cut of any considerable size; the others were merely punctured, and completely obstructed by the protrusion of the villous coat. The distention of flatus was enormous; strangulation being perfect. The bowel and omentum were carefully placed in a basin of warm water, cleansed and softened, (they had become completely glazed) the large wound was closed with the Glover's Suture, the small ones let alone. I then introduced a grooved director, and enlarged the wound in the abdominal walls about one-half; by gentle manipulation, returned the bowel the reverse of the manner of its escape, and finally the large quantity of omentum. The wound was brought together and confined, by means of two or three interrupted sutures, a compress and bandage being applied. I proceeded to examine various other incised and punctured injuries in different parts of the body. One was immediately over the liver, and penetrated the peritoneal coat; but as there was little or no hemorrhage, I presume it did not enter that organ. Finally the patients were given an additional opiate and allowed to rest. The next morning I called to see those patients, and to my surprise, I found both without a bad symptom. Reaction had come on, but not too much. The bowels were kept confined in S.'s case; antiphlogistic remedies recommended, and I saw no more of them, Dr. Peyton taking charge of the cases. Two or three weeks afterwards I learned that S. was at the County seat, Raleigh, ready for another bout with any man that crosses his path.

This case is reported to show the extraordinary recuperative powers of nature when backed by indomitable resolution and a will of iron; and furthermore, that we should never desert a case in Surgery, as long as there is the evidence of remaining vitality, and there is no vital part mortally wounded.—*Memphis Medical Recorder.*

---

Paris, June 30, 1856.

M. Baudens, Surgeon-in-chief of the French army in the East, has written a letter to the President of the Academie des Sciences, on the Typhus of the Crimea. He examines the differences between this typhus and the typhoid fever as it exists in France, and he concludes that these two diseases are essentially different one from the other; contagion, doubtful for typhoid fever, cannot be doubted for typhus. At the *Ambulance* of the first division of the army, almost all the Physicians and nurses, and nearly all the soldiers, admitted for other diseases, had typhus; fifteen Physicians out of sixteen had it. Between the Crimea and Constantinople, thirty-seven physicians, twenty sisters of charity, eight clergymen, many hundred nurses, all in full health, died from having been exposed to contagion. The incubation of the disease lasted about six days. The Secretary of Dr. Baudens was attacked seven days after having visited an Hospital. The causes of the typhus in the Crimea acquired a great power after the 1st of January, 1856, because then, the soldiers, on account of the cold, had shut themselves up in their tents, the ground of which was damp, and filled with impure matters.

A fact observed in France, upon soldiers coming from the Crimea, shows that the incubation of typhus may be much longer than the average fixed by Dr. Baudens. A regiment left Balaklava on the 29th of April, and came direct to Marseilles, where it landed on the 10th of May. From Marseilles it went up to Calais without any case of typhus; but, from thence, and after May 24, the regiment, travelling on foot, had to leave patients in all the places it passed through. At Neufchateau, ten soldiers attacked with typhus were left apparently very dangerously ill. Nevertheless, they all recovered, very likely because they were not exposed to the causes of the typhus, and were taken care of in every respect much better than the patients treated in the Crimea or at Constantinople. It results from this fact that the incubation of typhus may be longer than twenty-five days (from the 29th of April to the 24th of

May.) The details on this subject are related in a letter of M. Garcin, read at the Academie des Sciences on the 16th of June.

A very remarkable paper, on the power of adaptation of the eye to different distances, has been presented to the Academy, by M. Charles Rouget. After having given a new description of a part of the ciliary muscle, recently discovered by a pupil of Professor Donders, M. Rouget shows that the muscular fibres of the iris are a direct continuation of the fibres of this part of the ciliary muscle. He states also that the part already known of the ciliary muscle (*i. e.*, Bruecke's muscle) is in continuity on one side with the choroid, and, on the other side, with the membrane of Desceinet. These anatomical facts being well understood, it is easy to admit that a combined contraction of the ciliary muscle, and of the iris, will increase the antero-posterior diameter of the dioptrical apparatus of the eye. M. Rouget explains all the details of these phenomena, and also of many others, which, according to his anatomical researches, take place at the same time, and among these, particularly, a kind of erection of the iris, produced by the compression of its veins. In the last part of his paper, M. Rouget shows how the results of his researches agree with the important experiments of Krasmer, Helmholtz, and Donders.

An interesting discussion has taken place at the Academie de Medecine on the relations between the horse-disease called *grease* and small-pox. It is well known that Jenner thought that grease was the origin of cow-pox; although, in the only experiment he tried on this subject, he did not see cow-pox produced in a cow after the inoculation from the matter of grease. A case reported to the Academy by M. Bousquet seems to be favorable to the opinion of Jenner. A man having shod a horse attacked with grease, had, twenty-four days after, a vaccine eruption over his hands. The liquid of the pustules, inoculated to some children, produced true vaccine pustules. Another fact, almost of the same kind, was observed in Paris in 1805, on a driver. M. Leblanc, a learned veterinary surgeon, objects to the views of Jenner, and to the conclusions which M. Bousquet drew from the preceding facts. He relates many experiments, made by himself or by M. Bousquet, in which the liquid of grease, having been inoculated either in cows or in men, did not produce anything like vaccine. It seems that M. Leblanc, M. Bousquet, and some other Academicians, did not know fully the state of science on this subject. In an article published in the *Gazette Medicale de Paris*, (No. 24, 14th of June,) M. Chlozan shows that many experiments made by Lay, a physician of Pickering (Yorkshire,) and by Sacco, prove that in some circumstances the matter of grease may produce true cow-pox. These experimenters have also seen a few cases of production of vaccine in men who had been touching horses attacked with grease. Jenner relates two cases of this kind; and he says that the cases in which inoculation of grease to cows had produced no specific pustule, prove nothing, as it is well known that the inoculation of true cow-pox on a cow does not produce cow-pox.—*Letter to Med. Times and Gazette*, July, 1856.

